# Factor Structure and Measurement and Structural Invariance of the Edinburgh Postnatal Depression Scale during the Perinatal Period among Japanese Women: What Is the Best Model?

**DOI:** 10.3390/healthcare11121671

**Published:** 2023-06-06

**Authors:** Tomomi Saito, Kyoko Sakanashi, Tomoko Tanaka, Toshinori Kitamura

**Affiliations:** 1 Department of Obstetrics and Gynaecology, School of Medicine, Juntendo University, Tokyo 113-8421, Japan; 2 Kitamura Institute of Mental Health Tokyo, Tokyo 151-0063, Japan; 3 Kitamura KOKORO Clinic Mental Health, Tokyo 151-0063, Japan; 4 Department of Perinatal Mental Health of Aiiku Clinic, Aiiku Maternal and Child Health Center, Tokyo 105-8321, Japan; 5 Aiiku Research Institute for Maternal, Child Health and Welfare Imperial Gift Foundation Boshi-Aiiku-Kai, Tokyo 106-0047, Japan; 6 Graduate School of Health Sciences, Kumamoto University, Kumamoto 860-0811, Japan; 7 Kamoto Health Centre, Kikuchi 861-1306, Japan; 8 T. and F. Kitamura Foundation for Studies and Skill Advancement in Mental Health, Tokyo 151-0063, Japan; 9 Department of Psychiatry, Graduate School of Medicine, Nagoya University, Nagoya 464-8601, Japan

**Keywords:** perinatal depression, EPDS, factor structure, measurement invariance

## Abstract

The Edinburgh Postnatal Depression Scale (EPDS) is a widely used screening tool for perinatal depression. Its factor structure is still a debatable topic. Our study aimed to examine the factor structure and measurement invariances of the Japanese version of the EPDS from late pregnancy to early postpartum. A total of 633 women were followed with the EPDS at three times over the perinatal period: late pregnancy (*n* = 633), 5 days after childbirth (*n* = 445), and 1 month after childbirth (*n* = 392). We randomly divided the participants into two groups: one for exploratory factor analyses (EFAs) and another for confirmatory factor analyses (CFAs). The result of the EFAs indicated different factor models at each time point. Hence, CFAs were performed using the second sample set to compare different models including the ones previously reported. A 3-factor model consisting of depression (items 7, 9), anxiety (items 4, 5), and anhedonia (items 1, 2) (Kubota et al., 2018) was consistently stable during the whole perinatal period. Kubota’s 3-factor model showed invariance across the perinatal period.

## 1. Introduction

Depression during pregnancy and the postnatal period, known as perinatal depression, is a global health concern. In Japan, the incidence of antenatal/postnatal depression was 5.6%/5.0% [1]. Perinatal depression has been linked to a number of adverse child health and mother–child relationship outcomes [2,3]. Perinatal depression has also been associated with a variety of poor infant conditions including reduced cognitive [4], socio-emotional [5,6], and psychomotor development [7]. Early detection of perinatal depression is a very important clinical issue. The Edinburgh Postnatal Depression Scale (EPDS) [8], a 10-item self-administered questionnaire, is one of the screening instruments for postnatal depression. The EPDS is also used for an antenatal population [9,10]. The EPDS was translated worldwide, and its psychometric properties were examined in many countries including Japan [11].

### 1.1. EPDS: Factor Structure

Despite worldwide use, the EPDS has several weaknesses, one of which is an unclear factor structure and the lack of evidence of measurement invariances. Identifying latent factors of a screening instrument may help for different types of depression [12,13,14]. However, if a certain factor structure cannot be reliably reproduced, it is unlikely to be useful for these purposes. Unfortunately, previous factor analytic studies of the EPDS reported considerable variation, not just between but also within cultures.

Although the EPDS was developed as a unidimensional measure [8], unidimensionality could rarely be demonstrated [15]. There have been many studies of exploratory factor analysis (EFA), principal component analysis (PCA), and confirmatory factor analysis (CFA) of the EPDS. Kozinszky et al. [16] reviewed the past studies of EPDS factor structures and reported that most of them supported different types of 3-factor structure. They include 1-, 2-, and 3-factor structures. Most of them showed a 3-factor structure, details of which, however, considerably varied. Kozinszky et al. (2017) used ante- and postnatal samples (*n* = 2967 and 714, respectively) to which models of the EPDS factor structure from 42 previous reports were compared in terms of goodness of fit. They added models derived from their own EFAs. In addition, they created theory-driven models (TDMs) based on the literature and on neurobiological insight, which was previously published. First, they considered that items 1 (“unable to see the funny side”) and 2 (“not looked forward with enjoyment”) belonged to “anhedonia”; items 3 (“blamed myself”), 4 (“worried”), and 5 (“scared or panicky”) belonged to “anxiety”; items 8 (“sad and miserable”) and 9 (“unhappy and cried”) belonged to “low mood”; and items 6 (“things on top of me”) and 10 (“idea of harming myself”) belonged to “hopelessness”. Kozinszky et al.’s (2017) TDM1 consisted of “anhedonia”, “anxiety”, and “low mood”; TDM2 consisted of “anhedonia”, “anxiety”, and “hopelessness”; and TDM3 consisted of “anhedonia”, “anxiety”, “hopelessness”, and “low mood”. Considering that item 3 (“blamed myself”) was not an anxiety item but rather a part of “suicidal risk” with items 6 and 10, they modeled TDM4 as consisting of “anhedonia”, “anxiety” (without item 3), “low mood”, and “suicidal risk”. Even though three “suicidal risk” items were phenomenologically heterogeneous, Kozinszky et al. created a 3-factor model with “anhedonia”, “anxiety” (without item 3), and “low mood”: TDM5. Finally, TDM6 consisted of “anhedonia”, “anxiety”, and “suicidal risk” (Table 1). Kozinszky et al. compared the models derived from the previous studies, the model derived from their own EFA, and six TDMs in terms of goodness-of-fit indices (mainly chi-squared, comparative fit index (CFI), Tucker Lewis index (TLI), and root mean square of error approximation (RMSEA)) as well as Akaike information criteria (AIC). They prioritized AIC because the compared models were not nested ones. In both the ante- and postnatal samples, TDM5 was one of the best models. TDM6 was slightly superior to TDM5 among the postnatal sample, but it was inferior to TDM5 among the antenatal sample. Matthey’s (2008) model [17] showed a very low AIC in both ante- and postnatal samples, although their model consisted only of “anxiety” items. Brouwers et al.’s (2001) model [18] showed the lowest AIC among the postnatal samples, but its fit indices were virtually the same as those of TDM5 and worse among the antenatal sample.

An aim of the present study is to apply Kozinszky et al.’s (2017) model comparison approach to our perinatal study in Japan. We do not think that EFA is the most appropriate method to examine the factor structure of a psychological measure. Rather we believe that comparison of multiple models to a set of data will give us a wider perspective of the latent structure of the concept. This may also give us an insight about culturally specific aspects of its factor structure. 

### 1.2. EPDS: Measurement Invariance

Another important aspect of a psychological measure is its temporary stability. Although factor analyses of the EPDS have been investigated in many countries, most of these studies were cross-sectional and only examined it at a single time point. There have been few reports examining the stability of the factor structure over the perinatal period in the same sample [19,20,21]. Cunningham et al. (2015) [20] reported different factor structures on admission for psychiatric treatment and before leaving the hospital postpartum. In a large community sample, Swalm et al. (2010) [21] reported the same factor structures ante- and postpartum. Jomeen and Martin (2005) [22] showed that the factor structure of the EPDS differed depending on gestational weeks. For example, item 8 (“sad and miserable”) was included as a factor of anhedonia at 14 weeks of gestation, whereas it was included as a factor of anxiety at 27 to 40 weeks of gestation. If the factor structure differs across multiple time points (e.g., ante- and postnatal period), it makes no sense to compare the scores of the measure. Kubota et al. (2018) identified a 3-factor structure—anhedonia (items 1 and 2), anxiety (items 4 and 5), and depression (items 7 and 9)—and this model showed stability across three time points (late pregnancy, and 5 days and 1 month after childbirth). We presume that the factor structure of the EPDS may differ across the perinatal periods because of different biological and social conditions.

The best-fit model of the factor structure should be examined in terms of measurement invariance when used on different occasions. These procedures include [23]:

(a) Configural invariance: both groups (e.g., nulliparae multiparae) have the same indicators in each factor.
(b) Metric invariance; also known as weak factorial invariance: factor loadings of both groups are invariant for the corresponding indicators.
(c) Scalar invariance; also known as strong factorial invariance: intercepts of both groups are invariant for the corresponding items.
(d) Residual invariance; also known as strict factorial invariance: residuals of both groups are invariant for the corresponding items.

The conditions from (b) to (d) are termed measurement invariance because they are concerned about the relationships between measured indicators and their latent constructs. Testing hypotheses is recommended to be carried out in the above order [23]. Rejection of a particular step means that the subsequent steps are not allowed.

We think that when more than one model of the EPDS factor structure is virtually the same in goodness of fit at one time point, the one better in measurement invariance should be given priority. Therefore, the second aim of our study is to propose the final model’s measurement invariance across the perinatal period.

## 2. Materials and Methods

### 2.1. Study Procedures and Participants

This is a secondary analysis of a large community perinatal study. In 2011, the Kumamoto Prefectural Government conducted a longitudinal study on the perinatal mental health of a pregnant community of women through to their postnatal period. All 55 obstetric clinics in the prefecture were invited to participate in this follow-up survey. Eighteen (33%) antenatal institutes responded to this request. These included one university hospital, public and private hospitals (*n* = 12), and private clinics (*n* = 5). Hence, this was a mixture of different types of antenatal institutions. The entry criterion was women of at least 28 weeks’ gestation who attended one of these antenatal clinics during the entire month of November 2011. Eligibility criteria were pregnancy (of at least 28 weeks’ gestation) and over 20 years old. We excluded those women who were illiterate in Japanese, who had severe mental illness, or who had been hospitalized with pregnancy complications. Sets of questionnaires were distributed on three occasions: (a) during the third trimester of pregnancy (Wave 1), (b) five days after childbirth (Wave 2), and (c) one month after childbirth (Wave 3). There were 1453 eligible women. Of these, 633 (44%), 445 (31%), and 392 (27%) returned the questionnaires during the third trimester, and 5 days and 1 month after childbirth, respectively.

### 2.2. Measurements

*Depression:* We used the EPDS at all the observation times. This is a 10-item questionnaire rated on a 4-point scale (0 to 3) to assess postnatal depression and is commonly used in many perinatal settings. Excellent internal consistency (Cronbach’s alpha = 0.87) and reliability (split-half reliability = 0.88) were reported for the original English version. Okano et al. (1996) [11] translated the EPDS into Japan and verified it by back-translation. This version enjoyed good internal consistency (Cronbach’s alpha = 0.78) and test–retest reliability (Spearman correlation = 0.92).

### 2.3. Data Analysis

We randomly divided the whole sample into two parts: one was used for EFAs and another for CFAs. We used SPSS "case selection" for this procedure. Using the first group, we checked the factorability by the Kaiser–Meyer Olkin (KMO) index and Bartlett’s sphericity test [24]. The skewness of all the EPDS items was also examined. All the EPDS item scores showed positive skew. Therefore, a log transformation was conducted to achieve an approximate normality assumption. We next performed a series of EFAs by the maximum-likelihood method with PROMAX rotation from a 1-factor structure and, subsequently, models with an increasingly greater number of factors (i.e., 2-, 3-factor structures, and so on).

Next, we conducted CFAs in the second halved sample and compared these models in terms of goodness of fit. This was a cross-validation of the models [25,26,27]. The fit of models with the data was examined in terms of chi-squared (χ^2^), CFI, and RMSEA. According to conventional criteria, a good fit would be indicated by χ^2^/df < 3, CFI > 0.97, and RMSEA < 0.08 [28,29]. We also used the Akaike information criterion (AIC) [30], where a lower AIC was judged as being better.

The next step was measurement invariance across different occasions of the best-fit model identified. We compared the model in terms of Wave 1 vs. Wave 2 vs. Wave 3. The definition of invariance from one step to the next was either (a) a non-significant increase in χ^2^ for df of difference, (b) a decrease in the CFI less than 0.01, or (c) an increase in the RMSEA less than 0.01 [31,32]. All the analyses were conducted by IBM SPSS Statistics version 26.0 and IBM Amos version 27.0 (IBM Japan, Tokyo, Japan).

### 2.4. Ethical Considerations

This study approval was given by the Institutional Review Board (IRB) of Kumamoto University School of Medical Sciences No. 269. Written informed consent was provided from the participants when distributing the questionnaire.

## 3. Results

### 3.1. EFA

Means, SDs, and skewness of all the EPDS items (original version and after log transformation version) are shown in Table 2. The EPDS item scores were low in most items. Some of them had a skew > 2.0. Nevertheless, the log transformation of the scale items resulted in the noticeable reduction in skewness.

The results of the KMO index at Waves 1 to 3 were 0.844, 0.868, and 0.845, respectively, using the data after logarithmic transformation in the first sample set. Bartlett’s sphericity test showed a significant result (*p* < 0.001) at the three time points. The data were adequate to perform EFAs. A scree plot suggested a 3-factor structure. However, the idiosyncrasy of the number of the factors in EFAs suggested the calculation of factor loadings of EPDS items in 1-, 2-, 3-, and 4-factor structures (Table 3, Table 4 and Table 5). In the 1-factor structure, all the items showed factor loading >0.3 [33] at the three time points. In the 2-factor model, items 1 and 2 (reflecting loss of interest) were loaded on the second factor, whereas the first factor was loaded on all the remaining items at only Wave 1. On the other hand, these results were different at Waves 2 and 3. In the 3-factor structure model, we noticed that items of the first factor of the 2-factor model were divided into two factors. In the 3-factor model, items 3, 4, 5, 6, and 8 were loaded on the first factor. Items 7, 9, and 10 were loaded on the second factor. The last factor was loaded on items 1 and 2, reflecting the loss of interest, at Waves 1 and 2. On the other hand, these results were different at Wave 3. In the 4-factor model, the first factor of the 3-factor model was divided into two. In the 4-factor model, items 3, 4, 5, and 6 were loaded on the first factor. Items 7, 9, and 10 were loaded on the second factor. Items 1 and 2, reflecting the loss of interest, were loaded on the third factor. The last factor was loaded on items 8 at only Wave 1. On the other hand, these results were different at Waves 2 and 3 (Table 3, Table 4 and Table 5).

### 3.2. CFA

Having obtained 1-, 2-, 3-, and 4-factor models derived from EFAs, there were no factor structures with the same configuration (i.e., the same items were loaded on the same factor) at all three time points. Therefore, we used the second sample to conduct a comparison of the models proposed by previous reports including all the TDMs of Kozinszky et al. (2017) as well as two of Kubota’s models [34,35]. It was found that the lowest AIC was obtained for TDM5 and the Kubota 2018 model at every time point (Table 6). AICs were virtually the same for the two models. Therefore, we concluded that these two models have the possibility to describe the present data best. Subsequent analyses were conducted using these models.

#### Measurement Invariance between Different Observation Times

We then compared the TDM5 and Kubota 2018 models in terms of measurement invariance across the three observation times (Table 7). TDM5 was accepted up to the metric level but rejected at the scalar level. On the other hand, the Kubota 2018 model was accepted up to the scalar model but rejected at the residual level. Therefore, the Kubota 2018 model showed superiority to TDM5.

## 4. Discussion

In this study, we first endeavored to find the factor structures of the EPDS among antepartum and postpartum samples. This, however, revealed that EFA-derived models did not show configural invariance across the observation time points. We then compared models previously recommended as the best. Models were compared in terms of measurement invariance across the perinatal period. It was found that the Kubota 2018 model should be given priority. This is encouraging in using the Kubota 2018 model when comparing the EPDS scores at different time points in clinical as well as research settings. The Kubota 2018 factor structure was the same as that reported by Coates et al. (2017) [36] who examined the factor structure of the EPDS in pregnancy and postpartum at four time points, i.e., at 18 and 32 weeks gestation; and at 8 weeks and 8 months postpartum. Our study replicated Coates et al.’s (2017).

It is interesting that EPDS items 1 and 2 reflecting loss of interest often consist of one factor: the factor of anhedonia (e.g., [19,20]). It seems feasible to speculate discreteness of this category. Neurobiological evidence suggests that anhedonia defines a particular dimension in depressive disorder. Anhedonia is associated with disturbances of dopaminergic neurons [37]. On the other hand, differentiation between anxiety and depressed mood among perinatal women has been debatable (see review of Kozinszky et al. [16]). Among male and female populations, there is a consensus that anxiety and depression are discrete [38,39] but its boundaries have been debatable. We should also pay attention to items that were deleted from factor analyses. One such item is item 10 (“idea of harming myself”). This represents a desire to harm oneself: suicidality. Perinatal suicidality is an important health issue [40,41,42]. A Japanese study showed that 17% of women showed a wish to harm oneself at some time during the perinatal period [42]. EPDS item 10 was often dropped from factor analyses not because of lack of clinical importance but possibly because there is only one item tapping suicidal ideations and behaviors. To cast clinical light to perinatal suicidality, we should either (a) add a few items assessing suicidality to the EPDS or (b) make use of a measure specific to suicidality in addition to the EPDS.

There are limitations of this study. As a psychological measure, the EPDS, after more than three decades of worldwide use, should be tested against international standards of patient-reported measures such as PROMIS (2013) [43] and COSMIN [44]. We applied the EPDS at three time points, but there was only one prepartum time point. A further study may need more observation time points. An important issue is the lack of diagnostic specificity. Although the EPDS was originally developed as a screening tool of postnatal depression [8], there is no rigid correspondence of the EPDS items with those of the Major Depressive Episode. This may be the original authors’ idea to avoid from the screening instrument somatic symptoms (such as loss of or increase in appetite or body weight, and psychomotor retardation or agitation) that are not those of depression but of physical changes. As in cases of somatic illness or under chemotherapy, identification of depression among perinatal women is a very important diagnostic issue [45,46]. A recent study to identify the core symptoms of antenatal depression used both cluster analyses and item response theory and found that only two symptoms―loss of interest and low mood―were recognized as core symptoms [47]. It is important to pay attention to the anxiety category that was identified as a subscale of the EPDS. The perinatal period is a stage where different types of anxiety disorders are found including panic disorder and obsessive-compulsive disorder together with pregnancy- and childbirth-specific anxiety disorders such as tokophobia (e.g., [48]) and postnatal traumatic disorders [49]. The EPDS Anxiety subscale may be sensitive to such anxiety disorders. Another research issue is the cultural or linguistic influence on the factor structure. The factor structure of the EPDS was identified only in the Japanese language. The subscales identified in this study should be compared with those reported from other cultural or linguistic backgrounds. In addition, participants who were physically or mentally impaired may have dropped out of the study because of difficulty answering the questionnaire during the whole peripartum period. Inclusion of women with pregnancy or birth complications may cast a different light on the EPDS factor structure.

## 5. Conclusions

Despite these drawbacks, our study found that the factor structure of Kubota 2018 was invariant in terms of measurement occasions. This factor structure is consistently stable during the whole peripartum period. Interventions of perinatal depression should be provided in discrete phases and subtypes for perinatal women. Also of importance is the possible association of the EPDS subscale scores with different types of anxiety disorder during the perinatal period.

## Figures and Tables

**Table 1:** Theory-driven models (TDM 1 to 6) and Kubota models.

Models	Anhedonia	Anxiety	Low Mood	Hopelessness/Suicidality
Kozinszky TDM 1	1, 2	3, 4, 5	8, 9	
Kozinszky TDM 2	1, 2	3, 4, 5		6, 10
Kozinszky TDM 3	1, 2	3, 4, 5	8, 9	6, 10
Kozinszky TDM 4	1, 2	4, 5	8, 9	3, 6, 10
Kozinszky TDM 5	1, 2	4, 5	8, 9	
Kozinszky TDM 6	1, 2	4, 5		3, 6, 10
Kubota 2014	1, 2	3, 4, 5	7, 8. 9	
Kubota 2018	1, 2	4, 5	7, 9	

**Table 2:** Means, SDs, and skewness of EPDS items (original version and after log transformation version).

	Original						After Log Transformation					
	Pregnancy (*n* = 329)		5 Days after Childbirth (*n* = 237)		1 Month after Childbirth (*n* = 201)		Pregnancy (*n* = 329)		5 Days after Childbirth (*n* = 237)		1 Month after Childbirth (*n* = 201)	
EPDS Item	Mean (SD)	Skewness	Mean (SD)	Skewness	Mean (SD)	Skewness	Mean (SD)	Skewness	Mean (SD)	Skewness	Mean (SD)	Skewness
1	0.08 (0.29)	3.85	0.11 (0.37)	3.93	0.07 (0.29)	4.55	0.05 (0.19)	3.49	0.07 (0.23)	3.01	0.05 (0.18)	4.05
2	0.09 (0.29)	3.45	0.13 (0.38)	3.51	0.10 (0.32)	3.05	0.06 (0.19)	3.24	0.09 (0.24)	2.68	0.07 (0.22)	2.81
3	1.10 (0.97)	0.35	0.84 (1.00)	0.78	0.60 (0.83)	1.13	0.63 (0.49)	−0.15	0.47 (0.52)	0.41	0.36 (0.46)	0.71
4	1.18 (0.97)	0.10	0.89 (0.97)	0.53	0.68 (0.92)	0.99	0.67 (0.50)	−0.32	0.50 (0.52)	0.25	0.39 (0.49)	0.66
5	0.73 (0.84)	0.75	0.52 (0.79)	1.43	0.35 (0.68)	1.79	0.44 (0.47)	0.36	0.31 (0.44)	0.93	0.21 (0.39)	1.52
6	0.79 (0.77)	0.90	1.02 (0.82)	0.39	1.04 (0.83)	0.39	0.49 (0.42)	0.10	0.61 (0.43)	−0.28	0.62 (0.44)	−0.28
7	0.31 (0.61)	2.03	0.23 (0.51)	2.19	0.20 (0.51)	2.52	0.20 (0.36)	1.50	0.15 (0.32)	1.86	0.13 (0.31)	2.20
8	0.49 (0.72)	1.35	0.48 (0.76)	1.42	0.36 (0.63)	1.78	0.31 (0.42)	0.86	0.29 (0.43)	1.02	0.23 (0.37)	1.24
9	0.22 (0.48)	2.35	0.17 (0.48)	3.11	0.15 (0.47)	3.77	0.14 (0.30)	1.85	0.11 (0.28)	2.55	0.09 (0.27)	2.92
10	0.16 (0.47)	3.15	0.10 (0.37)	4.07	0.06 (0.29)	5.34	0.10 (0.28)	2.66	0.06 (0.22)	3.68	0.04 (0.18)	4.85

Range of item scores 0 to 3.

**Table 3:** EFA of the EPDS during pregnancy.

Item	1-Factor	2-Factor		3-Factor			4-Factor			
	I	I	II	I	II	III	I	II	III	IV
1	0.45	−0.10	0.99	−0.09	0.09	0.79	−0.08	0.14	0.67	0.04
2	0.46	0.05	0.72	0.04	−0.11	0.93	0.06	−0.10	1.04	−0.05
3	0.65	0.71	−0.05	0.74	0.02	−0.02	0.64	0.02	−0.02	0.12
4	0.60	0.65	−0.05	0.85	−0.16	0.01	0.75	−0.12	0.02	0.08
5	0.65	0.75	−0.12	0.72	0.08	−0.08	0.81	0.12	−0.03	−0.014
6	0.44	0.50	−0.09	0.26	0.24	−0.02	0.28	0.23	0.04	−0.03
7	0.77	0.70	0.11	0.21	0.57	0.07	0.14	0.48	0.05	0.20
8	0.82	0.76	0.10	0.47	0.33	0.12	0.06	−0.01	−0.01	0.97
9	0.73	0.64	0.13	−0.08	0.89	0.04	−0.06	0.79	0.07	0.08
10	0.61	0.57	0.05	−0.06	0.81	−0.10	0.02	0.85	−0.06	−0.13

**Table 4:** EFA of the EPDS at 5 days after childbirth.

Item	1-Factor	2-Factor		3-Factor			4-Factor			
	I	I	II	I	II	III	I	II	III	IV
1	0.46	0.18	0.33	−0.04	0.02	0.67	−0.06	0.03	0.68	0.04
2	0.42	0.23	0.22	−0.03	−0.12	0.78	0.00	−0.12	0.76	−0.02
3	0.67	0.63	0.09	0.65	0.07	0.00	0.17	0.02	0.02	0.81
4	0.70	0.92	−0.17	1.02	−0.16	−0.13	0.89	−0.13	−0.12	0.12
5	0.77	0.74	0.08	0.72	0.11	−0.00	0.70	0.09	−0.03	0.08
6	0.49	0.42	0.09	0.32	−0.01	0.25	0.29	−0.02	0.24	0.08
7	0.62	0.27	0.41	0.21	0.40	0.10	0.33	0.38	0.07	−0.10
8	0.83	0.62	0.27	0.54	0.19	0.19	0.69	0.17	0.17	−0.13
9	0.70	−0.04	0.93	−0.04	0.83	0.10	−0.12	0.86	0.11	0.10
10	0.55	−0.04	0.71	−0.04	0.87	−0.18	0.01	0.83	−0.17	−0.02

**Table 5:** EFA of the EPDS at 1 month after childbirth.

Item	1-Factor	2-Factor		3-Factor			4-Factor			
	I	I	II	I	II	III	I	II	III	IV
1	0.60	−0.19	0.98	0.04	0.86	−0.09	−0.04	0.95	−0.01	0.02
2	0.57	−0.03	0.73	−0.04	0.74	0.10	0.10	0.61	0.01	0.06
3	0.65	0.66	0.06	0.56	0.00	0.17	0.63	0.11	0.06	−0.03
4	0.65	0.71	0.02	0.55	−0.02	0.23	0.68	0.07	0.07	−0.06
5	0.52	0.77	−0.20	0.09	−0.04	0.92	0.76	−0.14	−0.09	0.19
6	0.42	0.44	0.02	0.32	0.02	0.16	0.48	0.07	−0.01	−0.11
7	0.65	0.27	0.46	0.46	0.29	−0.03	0.09	0.23	0.46	−0.07
8	0.77	0.65	0.18	0.92	−0.10	−0.04	0.26	−0.09	0.68	−0.03
9	0.74	0.37	0.44	0.65	0.22	−0.10	−0.10	0.04	0.83	0.09
10	0.42	0.21	0.28	−0.08	0.39	0.36	−0.02	0.05	0.03	0.98

**Table 6:** CFAs of the EPDS and the best model selected at each time point.

Models	χ^2^	df	χ^2^/df	CFI	RMSEA	AIC
Pregnancy						
Kozinszky TDM 1	15.260	11	1.39	0.993	0.023	63.260
Kozinszky TDM 2	11.989	11	1.09	0.998	0.011	59.989
Kozinszky TDM 3	IS	IS	IS	IS	IS	IS
Kozinszky TDM 4	IS	IS	IS	IS	IS	IS
Kozinszky TDM 5	11.322	6	1.89	0.990	0.035	53.322
Kozinszky TDM 6	33.468	11	3.04	0.953	0.053	81.468
Kubota 2014	29.974	17	1.76	0.982	0.032	83.974
Kubota 2018	11.482	6	1.91	0.989	0.035	53.482
5 days after childbirth						
Kozinszky TDM 1	27.846	11	2.53	0.969	0.045	75.846
Kozinszky TDM 2	IS	IS	IS	IS	IS	IS
Kozinszky TDM 3	IS	IS	IS	IS	IS	IS
Kozinszky TDM 4	32.087	21	1.53	0.983	0.027	98.087
Kozinszky TDM 5	11.524	6	1.92	0.988	0.035	**53.524**
Kozinszky TDM 6	8.876	11	0.81	1.000	0.000	56.876
Kubota 2014	46.459	17	2.73	0.957	0.048	100.459
Kubota 2018	18.046	6	3.01	0.975	0.052	60.046
1 month after childbirth						
Kozinszky TDM 1	28.731	11	2.61	0.933	0.047	76.731
Kozinszky TDM 2	IS	IS	IS	IS	IS	IS
Kozinszky TDM 3	IS	IS	IS	IS	IS	IS
Kozinszky TDM 4	39.886	21	1.9	0.941	0.035	105.886
Kozinszky TDM 5	19.037	6	3.17	0.929	0.054	**61.037**
Kozinszky TDM 6	17.462	11	1.59	0.968	0.028	65.462
Kubota 2014	33.992	17	2.00	0.942	0.037	87.992
Kubota 2018	15.914	6	2.65	0.920	0.047	**57.914**

**Table 7:** Measurement and structural invariance of the EPDS.

Model	χ2	df	χ2/df	Δχ2 (df)	CFI	ΔCFI	RMSEA	ΔRMSEA	AIC	Judgement
TDM 5										
Configural	55.915	18	3.106	Ref	0.982	Ref	0.021	Ref	181.915	ACCEPT
Metric	63.525	24	2.647	7.610 (6) NS	0.981	0.001	0.019	Δ0.002	177.525	ACCEPT
Scalar	160.742	36	4.465	97.217 (12) *	0.940	0.041	0.041	0.022	250.742	REJECT
KUBOTA 2018										
Configural	38.304	18	2.128	Ref	0.989	Ref	0.016	Ref	164.304	ACCEPT
Metric	48.055	24	2.002	9.751 (6) NS	0.987	0.002	0.015	Δ0.001	162.055	ACCEPT
Scalar	140.984	36	3.916	92.929 (12) *	0.942	0.045	0.025	0.010	230.984	ACCEPT
Residual	416.971	48	8.687	275.987 (12) *	0.797	0.145	0.041	0.016	482.971	REJECT

* *p* < 0.001, NS, not significant.

## Data Availability

Due to the nature of this research, participants of this study did not agree for their data to be publicly shared, so supporting data are not available.
